# Carboxymethylation and Evaluation of Cassava (*Manihot esculenta*) Starch as a Potential Super‐Disintegrant in Tablet Formulations

**DOI:** 10.1155/bmri/4575160

**Published:** 2026-07-11

**Authors:** Tsegay Abraha, Fantahun Molla, Amanuel Teklebrhan, Huria Hussen, Afewerk Getachew

**Affiliations:** ^1^ Department of Pharmaceutical Industry Development, Armauer Hansen Research Institute, Addis Ababa, Ethiopia, ahri.gov.et; ^2^ Department of Pharmaceutics and Social Pharmacy, School of Pharmacy, College of Health Sciences, Addis Ababa University, Addis Ababa, Ethiopia, aau.edu.et; ^3^ Department of Pharmaceutics, School of Pharmacy, College of Health Sciences, Mekelle University, Mekelle, Ethiopia, mu.edu.et; ^4^ Department of Pharmaceutics, School of Pharmacy, Asrat Woldeyes Health Science Campus, Debre Birhan University, Debre Berhan, Ethiopia, dbu.edu.et

**Keywords:** carboxymethylation, cassava starch, disintegrant, starch modification, tablets

## Abstract

**Introduction:**

Cassava (*Manihot esculenta*) is a significant source of starch in Ethiopia, with potential pharmaceutical applications. However, native starches often require modifications to enhance their utility, particularly as disintegrants. Carboxymethylation is a well‐known modification that improves swelling and disintegration capacity. Therefore, this study is aimed at evaluating carboxymethylated cassava starch (CMCS) as a tablet disintegrant.

**Methods:**

CMCSs were produced using monochloroacetic acid under varying reaction conditions. Structural modification was confirmed with FTIR spectroscopy. Physicochemical and powder properties, including degree of substitution (DS), swelling power, viscosity, and flow behavior, were characterized using standard methods. Tablets were prepared using the direct compression method. The tablets were assessed for physical characteristics, disintegration, and dissolution to compare the disintegrant performance of CMCS, native cassava starch (NCS), and sodium starch glycolate (SSG).

**Results:**

Nine CMCS batches were obtained from the modification process and confirmed by FTIR analysis. CMCSs showed improved characteristics such as hydration capacity, swelling power, and solubility compared to NCS. Specifically, CMCS7 (DS = 0.227) exhibited comparable flow, swelling, hydration, and viscosity to SSG. Thus, it was selected as an optimal batch to be used as a disintegrant. Tablets formulated with CMCS7 exhibited superior mechanical strength, low friability, and higher disintegration efficiency ratios compared to NCS while performing comparably to SSG. Tablets containing CMCS showed comparable disintegration to SSG and enhanced initial dissolution.

**Conclusion:**

CMCS demonstrated promising disintegrant properties and could serve as a potential alternative local super‐disintegrant in pharmaceutical tablet formulations.

## 1. Introduction

Conventional tablets are considered effective when they disintegrate rapidly and release their contents right away [1]. This process is facilitated with the aid of disintegrants, which assist in the breakup of tablets into smaller fragments [2]. Among various disintegrants, starch is one of the earliest and most commonly used in solid dosage forms [3]. It is commonly obtained from potato, corn, rice, and cassava, generally referred to as official starches in pharmacopeias [4–6]. Hence, cassava starch has been investigated in Ethiopia as a potential local source of pharmaceutical starch [7].

**Figure 1:**
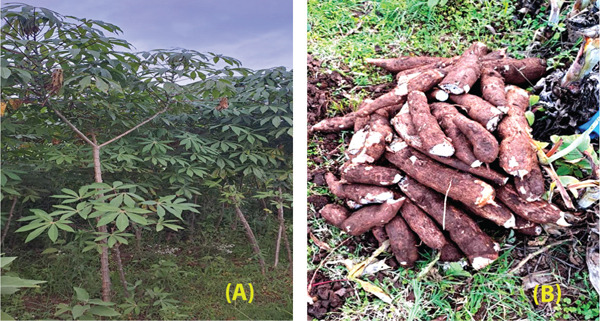
(A) Cassava plant in the field and (B) its tuber (image taken by Gulla A.).

Ethiopian cassava (*Manihot esculenta*) has been cultivated in the southern and western regions of the country, including Gamo Gofa, Illubabor, and Wollega [7–9]. The Ethiopian cassava plant and its tubers, dug out from the ground, are depicted in Figure 1 A and B, respectively. Starch from cassava tubers has been reported to exhibit higher swelling power (SP) as compared to corn, potato, and waxy corn starches [10]. Swelling is particularly vital for disintegrants, especially starches [10, 11]), which is directly associated with the amylopectin content [12, 13]. Ethiopian cassava starch contains relatively high amylopectin content (70.5%) [7], compared to yam (68.7), enset (70.24), and potato (57.2%) starches [14], hence indicating its strong potential as a local disintegrant in tablet formulations.

Although native starches are effective disintegrants, they often require high concentrations that may adversely affect key tablet properties such as flow, compressibility, hardness, and friability [15]. Thus, disintegrants that perform well at lower concentrations are preferred [16]. In addition, improved disintegration capacity becomes critical when rapid disintegration is needed. In this regard, modification is an area of interest to extend its utility and versatility [14, 17, 18]. Carboxymethylation is a simple, well‐established chemical modification that enhances the swelling and disintegration potential of starch ([19]; Noor Fadzlina et al., [20]). Importantly, carboxymethyl starch is officially listed in both the United States and British pharmacopeias [21, 22].

Carboxymethyl starch from various botanical origins has been studied for tablet disintegration, including *Cyperus esculentus* [23] and *Dioscorea hispida* [24] starch. However, the botanical origin of starch determines intrinsic characteristics such as amylose–amylopectin ratio, granule size distribution, crystallinity, and swelling behavior. Consequently, starches from different botanical sources may demonstrate differential reactivity during carboxymethylation and thereby exhibit distinct variations in physicochemical and functional performance [25]. Cassava‐based carboxymethyl starch has received comparatively limited attention, with only one study evaluating its application as a super‐disintegrant in fast‐dissolving domperidone tablets [26]. However, that investigation primarily emphasized formulation optimization and utilized a single degree of substitution (DS) (≈0.32), without examining the effects of reaction conditions or variations in DS on the resulting material properties and performance. Thus, the aim of this study was to produce and characterize Ethiopian cassava‐based carboxymethyl starch with varying DSs and to assess the best‐performing sample as a tablet super‐disintegrant.

## 2. Materials and Methods

### 2.1. Materials

Fresh Ethiopian cassava (*Manihot esculenta*) tubers were obtained from Areka Agricultural Research Institute, Areka City, Southern Nations, Nationalities and Peoples′ Region of Ethiopia. Hydrochlorothiazide (HCT) (China Chemical, China), magnesium stearate (BDH Chemicals Ltd., England), dicalcium phosphate (DCP) (Emcompress, Chemische Fabrik Budenheim, Germany), and Primojel sodium starch glycolate (SSG) (DFE Pharma, United States) were kindly donated by Addis Pharmaceutical Factory. Hydrochloric acid (Loba Chemie Pvt. Ltd., India), sulfuric acid (Sigma‐Aldrich, Germany), glacial acetic acid (Sigma‐Aldrich, Germany), and acetone (Sigma‐Aldrich, Germany) were kindly donated by the School of Pharmacy, College of Health Sciences, Mekelle University. Monochloroacetic acid (MCA) (Hopkin & Williams Ltd., England), sodium hydroxide (NeoLab, Life Science Co., India), ethanol (Fine Chemical General Trading, Ethiopia), methanol (Sigma‐Aldrich, Germany), sodium metabisulphite (Sisco Research Laboratories Pvt. Ltd., India), and sodium chloride (Blulux Laboratories Ltd., India) were purchased from the local market and used as received.

### 2.2. Methods

#### 2.2.1. Isolation of Starch From Cassava (*Manihot esculenta*) Tuber

Starch isolation was carried out following the method described by Abrha et al. [14]. First, the fresh tuber was cleaned, peeled, cut into small pieces, and blended using a blending machine (Sinbo, SHB‐3088, Philippines). The resulting paste was then suspended in distilled water containing 0.075% (*w*/*v*) sodium metabisulphite. Then, the suspension was passed through a fine muslin cloth, and the resulting filtrate was allowed to sediment. After that, the supernatant was decanted, and the sediment was repeatedly washed with distilled water until the supernatant became clear. The resulting starch was then dried in the open air at room temperature, milled to a fine powder, sieved (224‐*μ*m mesh sieve), and stored in an airtight container for further use.

#### 2.2.2. Preparation of Carboxymethylated Cassava Starch (CMCS)

CMCS with different DSs was prepared according to the procedures described by Yanli et al. [27], with minor modifications. First, a specific amount of NaOH was put into a round‐bottomed reactor flask equipped with a reflux condenser containing 80 mL of ethanol (80% *v*/*v*). The flask was then placed in an oil bath, maintained at a specific temperature, and continuously stirred until the NaOH was fully dissolved. The NCS (10 g) was then poured and vigorously mixed. Then, a specified amount of MCA was added, and the reaction was allowed to proceed for a specified time. The specific reaction conditions used are provided in Table 1. Subsequently, the flask was removed from the oil bath and cooled, and the pH of the slurry was adjusted to neutral using glacial acetic acid. The slurry was then filtered using filter paper (150 mm) and suspended in methanol. The suspension was filtered and repeatedly suspended in ethanol (80%) until the filtrate became negative for the silver nitrate test. The slurry was then dispersed in a sufficient quantity of acetone, stirred for 20 min, filtered, and dried in a hot air oven at 40°C for 48 h. The resulting solid mass was then ground, passed through a sieve (180 *μ*m), and stored for further use.

**Table 1:** Reaction conditions used for the carboxymethylation of cassava starch.

Code	nMCA/nAGU	nNaOH/nAGU	Reaction temp. (°C)	Reaction time (h)
Rxn1	0.5 (2.93)	1.5 (3.72)	50	1
Rxn2	1 (5.86)	1.5 (3.72)	50	1
Rxn3	1.5 (8.79)	1.5 (3.72)	50	1
Rxn4	1 (5.86)	2.5 (6.2)	50	1
Rxn5	1 (5.86)	2 (4.96)	50	1
Rxn6	1 (5.86)	1.5 (3.72)	50	3
Rxn7	1 (5.86)	1.5 (3.72)	50	2
Rxn8	1 (5.86)	1.5 (3.72)	30	1
Rxn9	1 (5.86)	1.5 (3.72)	40	1

*Note:* nMCA/nAGU, molar ratio of monochloroacetic acid to anhydrous glucose unit; nNaOH/nAGU, molar ratio of sodium hydroxide to anhydrous glucose unit.

#### 2.2.3. Physicochemical Characterization of the Starches

##### 2.2.3.1. DS

The DS was determined according to the method described by Nwokocha and Ogunmola [28]. CMCS (1 g) was treated with 10 mL of HCl (0.1 M in 80% methanol) for 1 h with occasional stirring. Then, it was continuously washed with 80% methanol until the filtrate became neutral in pH. The resulting sample was then dried in a hot‐air oven (Memmert, Germany) at 100°C for 1 h and allowed to cool in a desiccator. From this dried sample, 0.25 g was taken and put into a conical flask containing distilled water (100 mL) and 0.1 N NaOH (10 mL). The dispersion was then heated over a water bath for 20 min, and the hot solution was titrated with 0.1 N HCl solution using phenolphthalein indicator. The same procedure was performed on the NCS as a blank. Then, the percentage of carboxyl (*%* *C*) and DS were calculated using Equations (1) and (2), respectively:

(1) %C=Vb−VWt×M×4.5,

where *V*_*b*_ and *V* are the volumes of HCl used for the titration of the blank and the sample, respectively, *W*_*t*_ is the weight in grams of the sample, and *M* is the molarity of NaOH:

(2) DS=162×%C4500−58×%C.

Reaction efficiency (RE) was calculated from the DS and the reagent‐to‐starch molar ratio (RSMR), which is similar to MCA/AGU, using Equation (3) [29]:

(3) RE=DSRSMR×100.

##### 2.2.3.2. Moisture Content

Starch samples (2 g each) were weighed into dried Petri dishes and heated in an oven at 130°C for 2 h. The samples were then removed from the oven and weighed, and the moisture content was reported as a percentage weight difference [30].

##### 2.2.3.3. Hydration Capacity

First, a starch sample (1 g) and distilled water (10 mL) were placed in a centrifuge tube. Then, it was closed, shaken for 2 min, and allowed to stand for 10 min. Subsequently, it was centrifuged (at 1000 rpm) for 10 min, and the supernatant was decanted. Eventually, the weight of the wet mass was measured, and the hydration capacity was calculated as a ratio of the wet mass to the initial sample weight [31].

##### 2.2.3.4. Solubility (S) and SP

Starch samples (0.1 g each) were dispersed in distilled water (10 mL) in centrifuge tubes. The tubes were then kept in a water bath at 25°C, 37°C, 50°C, 60°C, 70°C, 80°C, and 90°C for 30 min, shaking every 5 min. Then, the suspensions were cooled and centrifuged for 15 min at 2000 rpm. The supernatants were decanted into Petri dishes and dried in an oven for 2 h at 120°C. The S and SP were then determined according to Equations (4) and (5), respectively [32]:

(4) S%=W1W3×100,

(5) SP=W2×100W3×100−S,

where *W*_1_ is the weight (g) of the soluble content in the supernatant, *W*_2_ is the precipitate weight (g), and *W*_3_ is the starch weight (g).

##### 2.2.3.5. Moisture Sorption Pattern

The moisture sorption pattern of NCS, CMCSs, and SSG samples was determined using the method described by Odeku and Picker‐Freyer [33]. First, starch samples were dried in an oven for 4 h at 120°C and spread evenly on dry, preweighed Petri dishes. The Petri dishes were then transferred to separate RH chambers (e.g., 100, 75.6, 60, 40, and 20 RH). The samples were left for 4 weeks to equilibrate at room temperature, and then their weights were measured and recorded. Next, the moisture absorption of each sample was calculated based on its weight difference at a given RH before and after equilibrium. The water sorption capacity was then reported as percent moisture uptake.

##### 2.2.3.6. Viscosity

The viscosity measurement was done according to the method described previously by Spychaj et al. [34]. A starch suspension (2% *w*/*v*) was made, and its viscosity was determined at 25°C using a rotational viscometer (Brookfield, RVDV‐II + Pro, United States) at 200 rpm using a spindle (No. RV‐02). The readings of viscosity were taken after 30 s of rotation.

#### 2.2.4. Characterization of Powder Properties

##### 2.2.4.1. Density‐Related Properties

To determine density‐related properties, 30 g of a sample (*W*_*s*_) was weighed and poured into a 250‐mL graduated measuring cylinder slowly at 45°. The bulk volume (*V*_*b*_) was determined after three taps on a wooden bench, 2 in. high. Then, it was placed on a tap densitometer (ERWEKA, SVM 223, Germany), and the taped volume (*V*_*t*_) was recorded after 250 taps. Finally, the bulk (*ρ*_*b*_) and tapped density (*ρ*_*t*_) were calculated using Equations (6) and (7), respectively:

(6) ρb=Ws/Vb,

(7) ρt=Ws/Vt.

##### 2.2.4.2. Flow Characterization

The flow rate and angle of repose were characterized using a powder flow tester (Pharma Test PTG‐S4, Germany). A preweighed mass of sample powder was filled into a stainless‐steel conical funnel with a 15‐mm outlet nozzle. Then, the machine was operated so that the sample powders flowed down a height of 10 cm onto a base of 10 cm in diameter, and a pile was formed. From this pile geometry, the angle of repose was automatically determined, and similarly, the mass flow rate was calculated from the known sample mass and the recorded discharge time.

Hausner′s ratio (HR) and Carr′s index (CI) were determined using Equations (8) and (9), respectively:

(8) HR=ρt/ρb,

(9) CI=ρt−ρbρt×100.

#### 2.2.5. Fourier‐Transform Infrared Spectroscopy (FTIR) Studies

FTIR spectra of the NCS and CMCS were obtained using an FTIR spectrophotometer (IRPrestige‐21, Shimadzu, Japan). First, the sample (8 mg) was mixed with a mulling agent (paraffin) and pressed between KBr plates. Then, the plates were placed in the FTIR, and the spectra were scanned over the wave number range of 4000–400 cm^−1^ with 20 scans and a resolution of 4 cm^−1^. The background spectrum was also collected before running the sample. The FTIR spectrum of CMCS was then compared with that of NCS for the characteristic peaks associated with the introduction of carboxymethyl groups into the starch molecules.

#### 2.2.6. Tablet Preparation

Tablets were prepared to evaluate and compare the efficiency of CMCSs with NCS and SSG as disintegrants. First, all the ingredients except magnesium stearate were sieved through a mesh (#40) and mixed using a laboratory‐scale mixer (MB005, Pharmatech, Buckinghamshire, United Kingdom) for 15 min. After that, magnesium stearate was added and mixed for 5 min. The powder blend was then directly compressed using a hydraulic tablet compressor (Riken Power, P‐16B‐027, China) with an 8‐mm flat‐face punch. Tablet compressions were made at a fixed die volume and target tablet weight of 250 mg. Different formulations were prepared using NCS, CMCS7, and SSG as disintegrants at different concentrations, as depicted in Table 2.

**Table 2:** Composition of different batches of hydrochlorothiazide tablets prepared.

Ingredients (%)	F1	F2	F3	F4	F5	F6	F7	F8	F9	F10	F11	F12	F13
HCT	20	20	20	20	20	20	20	20	20	20	20	20	20
NCS	1	2	3	4	—	—	—	—	—	—	—	—	—
CMCS7	—	—	—	—	1	2	3	4	—	—	—	—	—
SSG	—	—	—	—	—	—	—	—	1	2	3	4	—
MgS	0.25	0.25	0.25	0.25	0.25	0.25	0.25	0.25	0.25	0.25	0.25	0.25	0.25
DCP	q.s.	q.s.	q.s.	q.s.	q.s.	q.s.	q.s.	q.s.	q.s.	q.s.	q.s.	q.s.	q.s.

Abbreviations: CMCS7, Carboxymethylated Cassava Starch 7; DCP, dibasic calcium phosphate; HCT, hydrochlorothiazide; MgS, magnesium stearate; NCS, native cassava starch; q.s., quantity sufficient; SSG, sodium starch glycolate.

#### 2.2.7. Tablet Evaluations

##### 2.2.7.1. Tablet Dimensions and Hardness

Tablet thickness, diameter, and crushing strength (CS) were measured using a multipurpose hardness tester (Pharma Test, PTB 311E, D‐63512, Germany). Randomly selected, 10 tablets from each formulation were measured individually.

##### 2.2.7.2. Weight Variation

The weight variation test was determined by weighing 20 randomly selected tablets individually and calculating the average weight and standard deviation.

##### 2.2.7.3. Friability

Ten tablets from each batch were placed in a friability tester (Pharma Test, PTF 10E, Germany) and operated for 4 min at 25 rpm. The tablets were then collected, dedusted, and weighed, and the percentage weight loss was calculated and displayed as percentage friability.

##### 2.2.7.4. Disintegration Time (DT)

The DT was determined according to USP 30/NF 25 specification [22] using the USP disintegration tester (Pharma Test, PTZ S, D‐63512, Germany). In this case, six randomly selected tablets were placed in a disintegration apparatus containing 900 mL of distilled water and maintained at 37^°^C ± 2^°^C. The machine was then operated, and the time taken for complete disintegration was recorded as the DT. Then, the average DT and standard deviation of the six tablets were determined.

##### 2.2.7.5. Disintegration Efficiency Ratio (DER)

DER and the dimensionless comparator (DERc) were determined as in Equations (10) and (11), respectively [35]:

(10) DER=Cs/FtDt,

(11) DERc=DERsampleDERstandard,

where *C*_*s*_, *F*_*t*_, and *D*_*t*_ are the CS, friability, and DT, respectively.

##### 2.2.7.6. In Vitro Dissolution Study

The dissolution test was done according to the specification of USP 30/NF 25 [22] using a Dissolution Apparatus Type II (paddle) (Pharma Test, Hamburg, Germany) at a rotation speed of 50 rpm. First, six tablets from the selected batches were placed carefully in the dissolution vessel filled with 900 mL of 0.1 N HCl solution maintained at 37^°^C ± 0.5^°^C. Then, 10 mL aliquots of the dissolution medium were withdrawn at predetermined intervals, filtered using Whatman No. 1 filter paper, and appropriately diluted, and absorbance readings were taken with a UV/visible spectrophotometer (JENWAY, 6405, England) at 270 nm using 0.1 N HCl as a blank.

##### 2.2.7.7. Dissolution Efficiency (DE)

DE was obtained from the in vitro dissolution data using Equation (12) [36]:

(12) DE=∫t1tny.dt D100tn−t1×100,

where $\int_{t_{1}}^{t_{n}}y.\text{dt}$ is the area under the dissolution curve between time points *t*_1_ and *t*_*n*_, *y* is the percentage of drug dissolved at time *t*, and *D*_100_ is the 100% dissolution.

##### 2.2.7.8. Similarity Factor

Similarity factor (*f*_2_) was calculated from the in vitro dissolution data using Equation (13) [37]:

(13) f2=50log1001+1/n∑t=1nRt−Tt2,

where *n* is the number of time points, *R*_*t*_ is the dissolution profile of the reference product at time *t*, and *T*_*t*_ is the dissolution profile of the test product at time *t*.

#### 2.2.8. Data Analysis

Statistical analysis was performed using analysis of variance (ANOVA) with the statistical software (OriginPro 8.5 Corporation, United States). The Tukey multiple comparison test was used to compare the individual differences. At a 95% confidence interval, *p* values of ≤ 0.05 were considered statistically significant using the Tukey post hoc test. Tests were conducted in triplicate, and results are reported as mean and standard deviation.

## 3. Results and Discussions

### 3.1. FTIR Identification Studies

**Figure 2:**
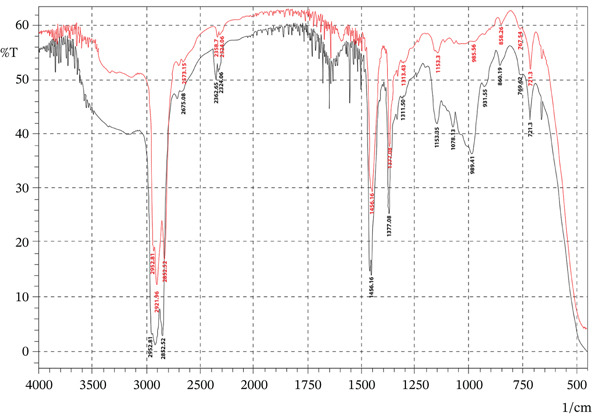
FTIR spectrum of native (black) and Carboxymethylated Cassava Starch 7 (red).

The infrared spectra of NCS and CMCS7 are presented in Figure 2. Accordingly, one of the prominent differences observed was the intense absorption band at around 1550–1700 cm^−1^. This peak is attributed to the stretching vibrations of the carboxylate groups (COO^−^) introduced during the reaction. The intensity of this peak can provide information about the DS, which refers to the number of carboxymethyl groups attached to each starch molecule [38]. Additionally, the FTIR spectra of CMCS show all typical peaks of starch with new additional changes in the peak intensities and positions associated with the hydroxyl groups (‐OH‐) due to the carboxymethylation reaction. The absorption band of –OH– was shifted and reduced in intensity for the CMCS. This shift and reduction of intensity in the absorption band might be due to the interaction of the ‐OH‐ group with ‐COO‐Na^+^ [23, 39, 40]. Typically, NCS exhibited characteristic peaks at 1078.13 and 1153.35 cm^−1^, known as the amylose peak, which corresponds to the stretching vibrations of the *α*‐(1‐4)‐glycosidic linkages in the amylose component of starch. However, these peaks changed intensity and position, attributed to the alterations in the crystallinity and molecular arrangement of the starch molecules, indicating that the CMCS sample was more amorphous than the native starch [38, 41].

### 3.2. DS

#### 3.2.1. The Effect of the MCA‐to‐Starch Ratio

**Figure 3:**
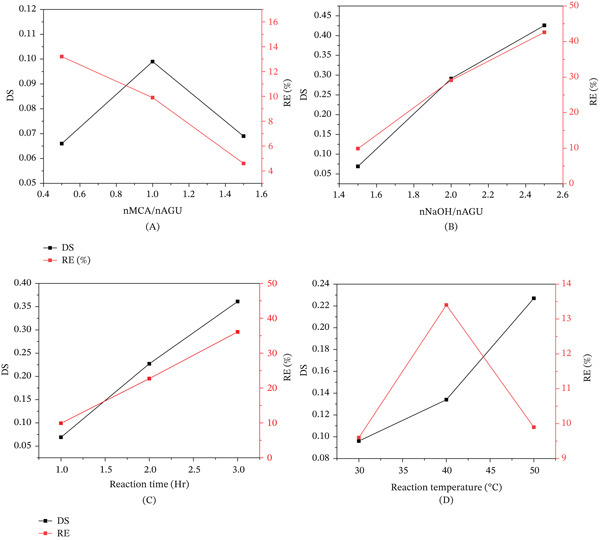
The influence of (A) nMCA/AGU, (B) NaOH/AGU, (C) reaction time, and (D) reaction temperature on the degree of substitution.

The influence of the molar ratio between MCA and starch on the DS is presented in Figure 3A. Accordingly, the DS increased significantly (*p* < 0.05) with an increasing MCA/AGU up to a 1.0 ratio, suggesting that etherification is favored within that range [42]. However, the DS decreased significantly (*p* < 0.05) at a higher MCA/AGU ratio (> 1.0), which could be attributed to the consumption of the NaOH by a side reaction. The more the MCA is, the less the NaOH can react with AGU, hence the lower DS [43]. This is consistent with a previous report on carboxymethyl Chinese yam starch [27].

#### 3.2.2. The Effect of the NaOH‐to‐AGU Ratio

The influence of various molar ratios of NaOH to starch on the DS is presented in Figure 3B. As depicted there, the initial increase in the molar ratio of NaOH/AGU favorably increased the DS until it reached 2.0. During the carboxymethylation process, the NaOH provides the alkaline environment for the reaction and serves as the swelling agent to facilitate the penetration of the MCA into the starch granules. A decrease in the slope of the increment in DS was observed as the NaOH ratio increased beyond 2.0. A possible reason for this observation could be that a further increase in NaOH dosage caused the inactivation of MCA as it was consumed in a side reaction [44]. The observation is in line with the findings reported in a study of carboxymethylation of *Borassus aethiopum* [38].

#### 3.2.3. The Effect of Reaction Time

As shown in Figure 3C, a significant increase (*p* < 0.05) in DS was observed as the duration of the reaction increased within the time frame studied (1–3 h), while the other conditions were kept constant. This result coincides with the findings of previous studies [27, 39]. The increase in DS could be attributed to the more effective interaction of the etherifying agent with the starch molecules, as the starch granules swell more with reaction time [45].

#### 3.2.4. The Effect of Temperature

The DS increased significantly (*p* < 0.05) with an increase in temperature from 30°C to 50°C, as depicted in Figure 3D. This could be due to the enhanced S of MCA, the swelling of the starch molecules, and the diffusion of the reactants as temperature increases, which in turn provides the highest reaction rate, leading to the highest DS values [39]. Temperatures exceeding 50°C were not applied to prevent gelatinization and destruction of the granular structure [46]. As determined by Defloor et al. [47], the average gelatinization onset temperature of different cassava varieties is 63.2°C. Thus, the optimum carboxymethylation temperature to obtain a high reaction rate without gelatinization is about 50°C.

### 3.3. Viscosity

**Figure 4:**
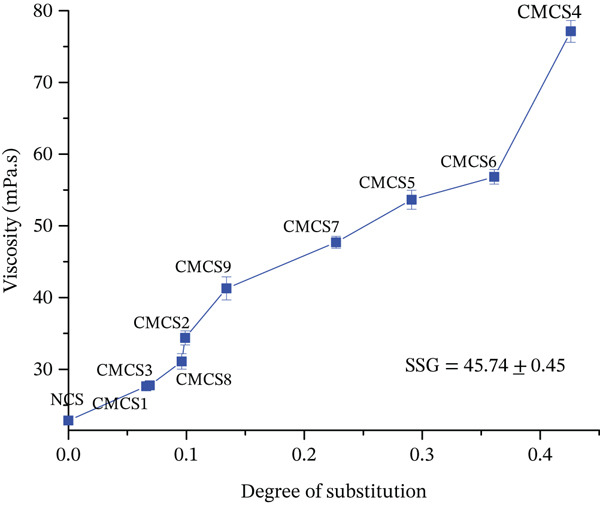
The effect of the degree of substitution on the viscosity of the starch samples.

The viscosities of CMCSs and SSG were statistically higher (*p* < 0.05) than those of the NCS, as depicted in Figure 4. The increase in the viscosity of the CMCSs may be due to the steric hindrance exhibited by the bulky carboxymethyl groups, which obstruct the proper alignment of the starch chain [48]. The larger molecular size of CMCSs might also be the reason for the increased viscosity [39]. The result is consistent with several studies [23, 48, 49]. However, Ganorkar and Kulkarni [50] reported that the incorporation of the carboxymethyl group resulted in a reduction of viscosity. This reduction in viscosity can be attributed to the aqueous–alkaline reaction medium, which primarily leads to partial depolymerization and molecular weight reduction [51]. Such chain degradation during etherification is known to directly influence viscosity [52]. In contrast, carboxymethylation carried out in hydroalcoholic media, as in the present study (80% ethanol), limits excessive granule swelling and minimizes alkaline chain scission, thereby preserving molecular weight [19]. Under such conditions, the introduction of hydrophilic carboxymethyl groups becomes the dominant factor. These groups enhance water binding and promote chain expansion via electrostatic repulsion, which increases the hydrodynamic volume and, consequently, the viscosity [53]. Hence, CMCSs with high DS could form a viscous gel mass when in contact with water. This might hinder the penetration of water into the tablet, hence retards tablet disintegration [49]. Among the modified starch samples, CMCS7 showed moderate viscosity, which was comparable with SSG.

### 3.4. Moisture Content and Moisture Sorption Pattern

**Figure 5:**
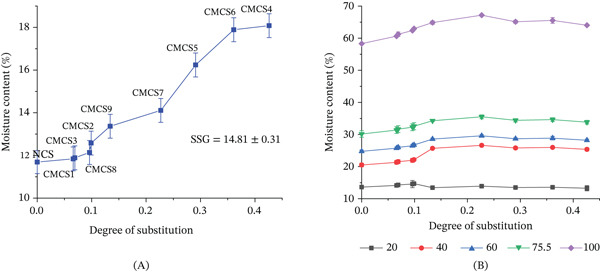
(A) Moisture content and (B) moisture sorption pattern of NCS and CMCSs (CMCS1–CMCS9).

Generally, CMCSs had higher moisture content compared to the NCS, as depicted in Figure 5A. CMCSs with a lower DS were observed to have lower moisture content than those with a higher DS. The increase in the moisture content might probably be attributed to the differences in the relative crystallinity and hydrophilic nature of the starches [54]. Moisture is known to modify the flow and mechanical properties of many powders [55]. The moisture content of air‐equilibrated roots and tuber starches ranges between 14% and 18% [56]. The maximum moisture content recommended for safe starch storage is 15% (*w*/*w*) [57, 58]. Generally, compared to SSG, most of the CMCSs had lower moisture contents except for CMCS4, CMCS5, and CMCS6.

The moisture sorption profiles of NCS, CMCSs, and SSG at different humidity levels are depicted in Figure 5B. The results showed that the percent moisture sorbed ranged from the lowest (13.26) at 20% RH for CMCS4 to the highest (69.7) at 100% RH for SSG. However, the moisture sorbed by CMCSs was significantly higher than that of NCS (*p* < 0.05) at all RH except 20%. This increased moisture sorption capacity of CMCSs promotes their swelling and hence the disintegration of CMCS tablets (Thoorens et al., [59]. Hence, these properties of CMCS powder can make it an excellent candidate to be used as a disintegrating agent.

### 3.5. Hydration Capacity, S, and SP

**Figure 6:**
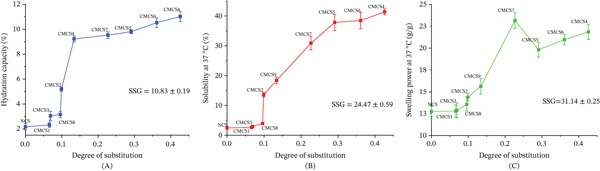
(A) Hydration capacity, (B) solubility, and (C) swelling power with respect to the degree of substitution at 37°C.

The hydration capacity of NCS, CMCSs, and SSG is depicted in Figure 6A. Statistically, the hydration capacity of CMCSs, except for CMCS1, was significantly higher than that of NCS (*p* < 0.05). These results correspond with carboxymethyl sweet potato starch [60]. The increase in hydration capacity implied that the modification might have weakened the associated forces in starch molecules [61]. It might also be attributed to the addition of the negatively charged carboxymethyl groups to the starch [23]. CMCS7 was found to have a comparable hydration capacity (9.53 ± 0.07) to SSG (10.83 ± 0.09).

The S and SP of all starches were generally low at low temperatures (25°C) but increased significantly at higher temperatures (90°C) (*p* < 0.05). This improvement due to temperature could be attributed to the increased mobility and dispersion of the starch in the solution [62]. However, the S and SP of the CMCSs showed a significant increment (*p* < 0.05) compared to the NCS. The finding agrees with the study on the S of CMCS [63]. For graphic comparison, the S and SP of NCS and CMCSs at 37°C are depicted in Figure 6B,C, respectively. Accordingly, the S and SP of most CMCSs, except for the lowest DSs, CMCS1, CMCS3, and CMCS6, were significantly higher than those of the NCS (*p* < 0.5). This might be due to the introduction of carboxymethyl groups, destruction of a helical structure, amylose loss [16, 42], loss of crystallinity, and increased amorphous region leading to more penetration of water, accompanied by the weakening of forces between the granules [40, 64]. Among the modified starches, CMCS7 exhibited the highest SP, which is somewhat closer to the reference, SSG. However, CMCSs with relatively higher DSs (4, 5, 6, and 7) showed significantly higher S compared to SSG (*p* < 0.5).

### 3.6. Powder Properties

Some powder properties of the NCS, CMCSs, and SSG are presented in Table 3. Bulk and tapped densities provide information on the flowability of powders [65]. Generally, the bulk and tapped densities decreased as DS increased. This could be attributed to the change in the granular structure/crystallinity of the powder during the modification, which could affect its powder properties [38]. The bulk density of the samples showed no significant difference compared to NCS (*p* > 0.05) except for SSG, CMCS1, CMCS7, and CMCS8. On the other hand, the tapped density of SSG, NCS, and CMCS7 demonstrated no significant difference. SSG, CMCS7, and CMCS9 showed lower CI than NCS and CMCSs with higher DS. According to CI, lower values, within the range 5–37, represent better flow properties than higher ones [66]. The foregoing also holds for the values of HR [67]. SSG and CMCS7 had the lowest HR, implying better flowability than NCS and CMCSs with higher DS.

**Table 3:** Powder properties of native and carboxymethylated cassava starches (mean [SD]).

Starch samples	Bulk density (g/cm^3^)	Tapped density (g/cm^3^)	Hausner′s ratio	Carr′s index (%)	Angle of repose (°)	Flow rate (g/sec)
SSG	0.67 (0.02)	0.77 (0.03)	1.15 (0.04)	11.79 (2.24)	26.95 (1.35)	9.73 (1.63)
NCS	0.49 (0.01)	0.62 (0.01)	1.27 (0.03)	20.96 (1.47)	^a^	^a^
CMCS1	0.62 (0.01)	0.78 (0.01)	1.25 (0.01)	20.51 (0.27)	^a^	^a^
CMCS3	0.51 (0.03)	0.65 (0.04)	1.27 (0.02)	20.46 (0.73)	^a^	^a^
CMCS8	0.55 (0.02)	0.81 (0.03)	1.46 (0.01)	31.81 (0.48)	^a^	^a^
CMCS2	0.49 (0.00)	0.61 (0.00)	1.24 (0.00)	19.56 (0.18)	28.45 (0.87)	4.41 (1.34)
CMCS9	0.48 (0.01)	0.59 (0.6)	1.24 (0.01)	19.55 (0.53)	26.24 (0.42)	5.82 (0.24)
CMCS7	0.37 (0.00)	0.43 (0.00)	1.16 (0.01)	13.17 (1.09)	23.42 (0.38)	7.97 (0.14)
CMCS5	0.43 (0.03)	0.54 (0.04)	1.25 (0.02)	19.80 (1.36)	30.47 (1.15)	2.5 (0.26)
CMCS6	0.46 (0.02)	0.64 (0.03)	1.37 (0.06)	28.52 (0.92)	^a^	^a^
CMCS4	0.36 (0.02)	0.62 (0.02)	1.71 (0.05)	41.65 (1.84)	^a^	^a^

*Note:* CMCS1–CMCS9, carboxymethylated cassava starches with different DS (0.066–0.426).

Abbreviations: NCS, native cassava starch; SSG, sodium starch glycolate.

^a^Undefined results.

As a general guide, powders with AR greater than 50° have unsatisfactory flow properties, whereas powders with AR close to 25°correspond to good flow (Amalia Yunia [68]). SSG, CMCS7, and CMCS9 had AR of 26.95, 23.42, and 26.24, respectively. The AR and flow rate for most samples were not determined as the powders did not flow through the opening of the measuring instrument. Therefore, these results are reported as undefined results (^a^) in Table 3. CMCSs with a DS greater than 0.3 showed poor flow properties, whereas those with a lower DS demonstrated improved flow properties.

### 3.7. Tablet Characterizations

#### 3.7.1. Tablet Thickness and Weight Uniformity

The weight variation and thickness uniformity of HCT tablets at different concentrations of NCS, CMCS7, and SSG as a disintegrant are summarized in Table 4. All the tablets were within an acceptable range of weight variation (±5%); the percentage deviation allowed for tablets weighing 250 mg or more [21]. Since DCP exhibited good flowability, incorporating a small amount of disintegrants did not affect the flowability of the blend; hence, good uniformity of tablet weight was obtained. Similarly, the mean tablet thickness and diameter of all the formulations did not show any significant variation (*p* > 0.05).

**Table 4:** Weight and thickness of HCT tablets formulated by direct compression with different disintegrant concentrations.

DC (% *w*/*w*)	NCS		CMCS7		SSG	
	Weight (mg)	Thickness (mm)	Weight (mg)	Thickness (mm)	Weight (mg)	Thickness (mm)
1	250.4 ± 1.85	3.24 ± 0.14	248.5 ± 4.18	3.38 ± 0.03	248.5 ± 2.94	3.42 ± 0.02
2	250.7 ± 3.07	3.45 ± 0.09	248.9 ± 2.34	3.37 ± 0.06	245.8 ± 3.63	3.34 ± 0.18
3	248.5 ± 2.42	3.44 ± 0.21	249.2 ± 2.18	3.40 ± 0.11	249.2 ± 1.33	3.49 ± 0.24
4	249.4 ± 3.07	3.45 ± 0.15	250.1 ± 2.84	3.42 ± 0.25	248.4 ± 1.93	3.40 ± 0.07

*Note:* DC (% *w*/*w*) refers to the disintegrant composition in the tablet formulations (% *w*/*w*).

Abbreviations: CMCS7, carboxymethylated cassava starch 7; NCS, native cassava starch; SSG, sodium starch glycolate.

#### 3.7.2. Hardness and Friability of HCT Tablets

**Figure 7:**
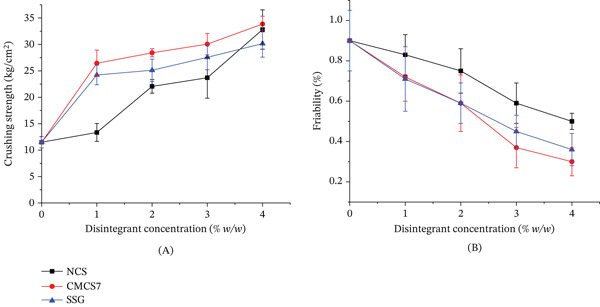
Effect of the concentration of NCS, CMCS7, and SSG employed as disintegrant on the (A) tablet hardness and (B) friability.

The CS of tablets prepared with different types and concentrations of disintegrants is shown in Figure 7A. Accordingly, tablets containing CMCS7 showed a superior degree of hardness compared to the others. Generally, the CS of all the tablets was well above the minimum requirement for a satisfactory tablet, about 4 kg [64]. The CS increased with the disintegrant concentration in the studied range, which is consistent with the result reported by Nattapulwat et al. [16]. This might be because starch‐based disintegrants could also possess a binding capacity [69, 70]. Plastic deformation of starch‐based materials enhances the overall plasticity of powder blends, promoting closer particle packing and stronger interparticle bonding [71]. Similarly, as depicted in Figure 7B, the friability decreased with the starch concentration in the range studied in all formulations. This was in agreement with the increase in CS, where, with higher CS, lower friability was observed. Tablets containing NCS were less hard with higher friability compared to CMCS7 and SSG tablets. Even so, the results always remained less than 1%, which is the maximum weight loss allowed according to USP 30/NF 25 [22].

#### 3.7.3. DT and DER

**Figure 8:**
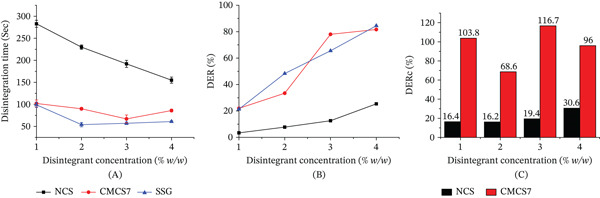
(A–C) Effect of concentrations of NCS, CMCS7, and SSG as disintegrants on disintegration time of the formulated hydrochlorothiazide tablets.

The effect of NCS, CMCS7, and SSG concentration on the DT of the tablets is shown in Figure 8A. Generally, all the formulations, except the formulation without a disintegrant (F13), passed the official disintegration test for uncoated tablets [21]. However, all the tablets formulated using CMCS7 showed significantly lower DT than NCS (*p* < 0.05). This could be explained by the higher SP of CMCS7 as a result of the modification [16]. A slight increase in DT was observed for CMCS7 and SSG at concentrations exceeding 3% and 2%, respectively. This is in line with the findings of a study on sodium carboxymethyl yam starch [16]. This is in line with the findings of a study on sodium carboxymethyl yam starch [16]. The observed trend can be attributed to the balance between capillary wicking and viscosity development in hydrophilic polymer systems. At low polymer concentrations, efficient capillary water uptake promotes rapid tablet wetting and disintegration [72]. However, increasing polymer loading can generate a more viscous microenvironment that restricts water penetration and delays tablet breakup [72, 74]. The slightly lower DT obtained for tablets formulated with SSG compared to CMCS7 might be attributed to the difference in modification and the source of the starch.

Comparison of DT is difficult, while there are variations in other parameters, such as hardness and friability. For that reason, DER has been used effectively as a better index that also evaluates the negative effect of these parameters on the DT of the tablet [35]. As depicted in Figure 8B, the general trend showed that the DER of SSG, CMCS7, and NCS increased with concentration. Specifically, CMCS7 showed significantly higher DER than NCS at all concentrations (*p* < 0.05). Higher DER values, in general, reflect better performance, indicating an optimal balance between rapid disintegration and sufficient mechanical strength in tablets [75]. Moreover, CMCS7 showed roughly comparable DER with SSG, except at 2% and 3%, where it showed lower and higher DER, respectively. This was similarly reflected in the DERc values, as depicted in Figure 8C for NCS and CMCS7 at different concentrations. DERc is a dimensionless parameter that provides a qualitative assessment of disintegrants′ performance on the overall tablet quality compared to a standard disintegrant [35]. Accordingly, CMCS7 showed significantly higher values compared to the NCS (*p* < 0.05), with three‐to‐sixfold performance over the concentration range studied. Moreover, CMCS7 showed comparable performance at 1% and 4%, while at 2% and 3% concentrations, its performance was 68.6% and 116.7% of the SSG performance, respectively.

#### 3.7.4. In Vitro Dissolution and DE

**Figure 9:**
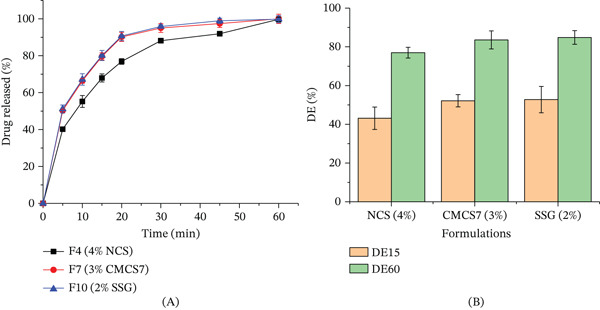
(A) In vitro dissolution profile and (B) dissolution efficiency of HCT tablets prepared from different concentrations of NCS, CMCS7, and SSG as a disintegrant.

The dissolution profiles of tablets containing NCS, CMCS7, and SSG as disintegrants at their best‐performing concentration are depicted in Figure 9A. The findings show that the cumulative drug release at 15, 30, and 45 min was significantly higher for tablets containing CMCS7 compared to NCS (*p* < 0.05). Moreover, CMCS7 demonstrated comparable efficiency to SSG (*p* > 0.05). The percentage of drug released at 20 min from tablets containing 3% disintegrants was in the order of CMCS7 (90.17 ± 2.42) > SSG (84.23 ± 3.24) > NCS (72.84 ± 2.08) (*p* < 0.05). At 30 min, the order was SSG (95.85 ± 3.91) > CMCS7 (92.81 ± 3.26) > NCS (83.52 ± 2.84). Overall, the percentage of drug release for all formulations was greater than 80% after 30 min; therefore, all formulations passed the dissolution test. However, DE provides a more comprehensive, meaningful, and mathematically sound measure of dissolution profile, making comparison easier and more robust [36]. Thus, the DE of the tablets formulated using NCS (4), CMCS7 (3), and SSG (2%) at their best‐performing concentration is depicted in Figure 9B. The order of the DE both at 15 and 60 min is SSG > CMCS7 > NCS. CMCS7 showed significantly improved DE compared to the NCS (*p* < 0.05). This confirmed that the modification enhanced both the early and late DE. Moreover, CMCS7 performed comparably to SSG (*p* < 0.05), as demonstrated by *f*_2_ values greater than 50 across all tested concentrations. An *f*_2_ value ≥ 50 indicates similarity between two profiles under specified conditions [37]. Specifically, the *f*_2_ value between CMCS7 (F7) and SSG (F10) was 94.8, indicating an excellent similarity and nearly superimposable dissolution profiles, as clearly depicted in Figure 9B.

## 4. Conclusion

CMCSs exhibited improved hydration capacity, S, and SP compared to NCS. Specifically, CMCS7 (DS = 0.227) demonstrated the most desirable balance of flowability, compressibility, and swelling properties. When used as a disintegrant in tablet formulations, CMCS7 produced tablets with higher mechanical strength, lower DTs, and rapid drug release compared to NCS. Moreover, these performances were comparable to a commercially available disintegrant, SSG. Therefore, CMCSs with low DS, such as CMCS7, could be a promising and local alternative super disintegrant in tablet formulations.

## Author Contributions

Tsegay Abraha, Fantahun Molla, and Afewerk Getachew conceived and designed the experiment. Tsegay Abraha performed the experiments. Tsegay Abraha, Fantahun Molla, Afewerk Getachew, Amanuel Teklebrhan, and Huria Hussen analyzed the data. Tsegay Abraha and Afewerk Getachew wrote the original paper. Amanuel Teklebrhan and Huria Hussen reviewed the original paper.

## Funding

This work was supported by Mekelle University, Mekelle, Ethiopia.

## Disclosure

All authors have read and approved the final manuscript. The research was conducted impartially, and there are no financial or personal relationships that could have influenced the results or the interpretation of the results.

## Conflicts of Interest

The authors declare no conflicts of interest.

## Data Availability

The article contains all the data necessary to support the findings of this study.
